# Genome-wide association study identifies glutamate ionotropic receptor *GRIA4* as a risk gene for comorbid nicotine dependence and major depression

**DOI:** 10.1038/s41398-018-0258-8

**Published:** 2018-10-04

**Authors:** Hang Zhou, Zhongshan Cheng, Nicholas Bass, John H. Krystal, Lindsay A. Farrer, Henry R. Kranzler, Joel Gelernter

**Affiliations:** 1 Department of Psychiatry, Yale University School of Medicine, New Haven, CT USA; 2 Molecular Psychiatry Laboratory, Division of Psychiatry, University College London, London, UK; 3 Department of Neuroscience, Yale University School of Medicine, New Haven, CT USA; 4 Clinical Neurosciences Division, VA National Center for PTSD, VA CT Healthcare System, West Haven, CT USA; 5 Department of Medicine (Biomedical Genetics), Boston University School of Medicine, Boston, MA USA; 6 Department of Neurology, Boston University School of Medicine, Boston, MA USA; 7 Department of Ophthalmology, Boston University School of Medicine, Boston, MA USA; 8 Department of Genetics and Genomics, Boston University School of Medicine, Boston, MA USA; 9 Department of Epidemiology and Biostatistics, Boston University School of Public Health, Boston, MA USA; 10 Department of Psychiatry, University of Pennsylvania Perelman School of Medicine, Philadelphia, PA USA; 11 VISN 4 MIRECC, Crescenz VA Medical Center, Philadelphia, PA USA; 12 Department of Genetics, Yale University School of Medicine, New Haven, CT USA; 13 Department of Psychiatry, VA CT Healthcare Center, West Haven, CT USA

## Abstract

Smoking and major depression frequently co-occur, at least in part due to shared genetic risk. However, the nature of the shared genetic basis is poorly understood. To detect genetic risk variants for comorbid nicotine dependence (ND) and major depression (MD), we conducted genome-wide association study (GWAS) in two samples of African-American participants (Yale-Penn 1 and 2) using linear mixed model, followed by meta-analysis. 3724 nicotine-exposed subjects were analyzed: 2596 from Yale-Penn-1 and 1128 from Yale-Penn-2. Continuous measures (*Fagerström Test for Nicotine Dependence* (FTND) scores and *DSM-IV* MD criteria) rather than disorder status were used to maximize the power of the GWAS. Genotypes were ascertained using the Illumina HumanOmni1-Quad array (Yale-Penn-1 sample) or the Illumina HumanCore Exome array (Yale-Penn-2 sample), followed by imputation based on the 1000 Genomes reference panel. An intronic variant at the *GRIA4* locus, rs68081839, was significantly associated with ND–MD comorbidity (*β* = 0.69 [95% CI, 0.43–0.89], *P* = 1.53 × 10^−8^). *GRIA4* encodes an AMPA-sensitive glutamate receptor that mediates fast excitatory synaptic transmission and neuroplasticity. Conditional analyses revealed that the association was explained jointly by both traits. Enrichment analysis showed that the top risk genes and genes co-expressed with *GRIA4* are enriched in cell adhesion, calcium ion binding, and synapses. They also have enriched expression in the brain and they have been implicated in the risk for other neuropsychiatric disorders. Further research is needed to determine the replicability of these findings and to identify the biological mechanisms through which genetic risk for each condition is conveyed.

## Introduction

Substance use is highly associated with other psychiatric illnesses^1–5^. For instance, substance use disorders (SUDs) and major depression (MD) are highly comorbid in the general population^2,3^, and strong associations between alcohol misuse and other psychiatric disorders were observed in a U.S. Army cohort^1^. Clinical outcome is worse in the patients with comorbid psychiatric disorders and SUDs than in each disorder separately^6^. The causes of this comorbidity are poorly understood, and a better understanding of the causal relationship and etiology may provide opportunities for risk mitigation. In recent years, genetic associations (pleiotropy) between specific substance use and psychiatric disorders have been investigated by genome-wide approaches^7–9^ and some specific genome-wide significant (GWS) loci that affect SUD/psychiatric comorbidity have been identified in our previous study^9^.

The association between cigarette smoking and MD is a particularly well-studied comorbidity, with several epidemiological studies showing co-occurrence^3,10–12^. Smoking initiation, daily smoking, persistent daily smoking, and heavy smoking were significantly associated with increased risk of MD, and the association also applies to nicotine dependence (ND)^13–15^. Different hypotheses have been proposed to explain the association. It has been suggested that depression may result from the neuropharmacological effects of nicotine or nicotine withdrawal^12,16,17^, or alternatively, that depression may cause smoking as an attempt at self-medication of negative feelings^18,19^ or that there are bidirectional causal effects linking smoking and depression^15^. Genetic risk variants for ND (as well as smoking-related behaviors) and MD (as well as depressive symptoms) have been separately identified in large cohorts by genome-wide association study (GWAS)^20–25^. Common risk factors or shared etiology for smoking and depression have also been suggested^26,27^, and genetic factors that predispose to both smoking and MD were also suggested in a study of female twins^28^.

To detect shared genetic variants that predispose to comorbid ND and MD, we conducted GWAS and meta-analysis on criterion counts comprised of *Fagerström Test for Nicotine Dependence* (FTND) scores and *DSM-IV* MD criteria in two African-American samples. A variant in *GRIA4*, the gene that codes for the subunit 4 of the α-amino-3-hydroxy-5-methyl-4-isoxazolepropionic acid (AMPA) glutamate receptor, showed genome-wide significance for association with ND–MD comorbidity.

## Materials and methods

### Participants and diagnostic procedures

A total of 4944 African American (AA) subjects were recruited for the Yale-Penn genetics of substance dependence study from 2000 to 2013, as previously described^22,29^. The subjects were grouped into two sets, Yale-Penn-1 (3227) and Yale-Penn-2 (1717), based on their epoch of recruitment and the genotyping platforms used. All subjects provided written informed consent, and certificates of confidentiality were obtained from National Institute on Drug Abuse (NIDA) and National Institute on Alcohol Abuse and Alcoholism (NIAAA). All subjects were interviewed using the Semi-Structured Assessment for Drug Dependence and Alcoholism (SSADDA)^30^. Lifetime FTND scores^31^ and criterion counts for MD from the *DSM-IV* (Diagnostic and Statistical Manual of Mental Disorders, Fourth Edition)^32^ were derived. Six items were assessed for the FTND, generating scores from 0 to 10 (the higher the score, the more severe the nicotine use), and nine criteria were assessed for MD, generating scores from 0 to 9. We scaled the FTND scores uniformly using the same range as for MD criteria so as to weight them comparably for the GWAS^9^. Then, the comorbid (summed) criterion counts (ranging from 0 to 18) were treated as the outcomes, representing the overall severity of comorbidity. Subjects who were not exposed to tobacco (i.e., who answered “no” to the question, *have you ever tried any form of tobacco?*) were excluded from the 4994 participants, leaving 3724 eligible subjects, 2596 from Yale-Penn-1 and 1128 from Yale-Penn-2.

### Genotyping, quality control, and imputation

The Yale-Penn-1 sample was genotyped using the Illumina HumanOmni1-Quad array containing ~988,000 SNPs. The Yale-Penn-2 sample was genotyped using the Illumina HumanCore Exome array containing ~266,000 exonic SNPs and ~240,000 tagging SNPs for genome-wide imputation. Individuals and SNPs with genotype call rates <98%, and SNPs with minor allele frequency (MAF) <1% were removed from downstream analyses. Yale-Penn-1 and Yale-Penn-2 data were analyzed separately.

To correct any misclassification from self-reported race, we conducted principal component (PC) analysis^33^ on SNPs common to both the two Yale-Penn genotype datasets and the 1000 Genomes phase 3 reference panel which contains African, American, Asian, and European populations^34^. SNPs were pruned based on LD (*r*^2^ < 0.2) using PLINK^35^. Yale-Penn subjects were clustered into different groups by the Euclidean distances to the reference populations (based on the first 3 PCs). For this study, subjects that clustered with non-African populations were removed from the downstream analyses. We then conducted a second PC analysis within the remaining Yale-Penn subjects and removed any outliers beyond three standard deviations from the mean. The first 10 PCs were used in all subsequent analyses to correct for residual population stratification.

We imputed additional single nucleotide variants (SNVs) using Minimac3 implemented in Michigan Imputation Server (https://imputationserver.sph.umich.edu/index.html)^36^ based on the 1000 Genomes phase 3 reference panel^34^. SNVs with Hardy–Weinberg equilibrium *P* values <10^−5^, imputation accuracy <0.8, or MAF <1% were excluded from downstream analyses. In the Yale-Penn-1 sample, 14,778,319 SNVs were included in the association analyses; in the Yale-Penn-2 sample, 9,658,251 SNVs were analyzed. 9,520,174 SNPs common in two samples were meta-analyzed.

### Phenotype imputation

ND scores or the set of MD criteria were incomplete in a small proportion of the sample: 4.7% (121) of the Yale-Penn-1 and 4.6% (52) of the Yale-Penn-2 subjects. To address this without the power reduction that would result from simply excluding these subjects, we used PHENIX^37^, a variational Bayesian method fitting in a Bayesian multiple-phenotype mixed model, to impute the missing criteria. ND and MD were imputed separately in the two datasets, using the correlation matrix of the subjects derived from genome-wide efficient mixed model association (GEMMA)^38^.

### Statistical analysis

We performed association tests for the ND+MD criterion counts (ranging from 0 to 18). All SNVs, both genotyped and imputed, were tested using a linear mixed model (GEMMA), adjusted by age, sex, and the first 10 PCs. Analyses were performed separately within each dataset. The association results were meta-analyzed across the two datasets, using the inverse variance method implemented in the program METAL^39^. Regional associations were plotted using LocusZoom^40^.

### Functional annotation and enrichment analysis

Functional annotations for the top variants and genes were explored from the literature and from expression databases, including Gene-Tissue Expression (GTEx, https://www.gtexportal.org/home/) for gene-tissue expression^41^ and BrainSpan (http://www.brainspan.org/) for information regarding the transcriptome across human brain development^42^. Genes co-expressed with the target gene were identified using COXPRESdb v6.0 (http://coxpresdb.jp/)^43^, a coexpression database of DNA-microarray and RNAseq-based expression data. Disease enrichment among the co-expressed genes was assessed using WebGestalt (http://www.webgestalt.org/option.php)^44^, a functional enrichment analysis web tool. Gene ontology (GO) enrichment of the genes mapped to the top SNVs (*P*-value < 1 × 10^−4^ in either dataset or meta-analysis) was analyzed using the web-based gene set analysis tool Gorilla (http://cbl-gorilla.cs.technion.ac.il/)^45^. Terms with false discovery rate (FDR) <0.05 were considered to be significantly enriched.

## Results

In total, 3724 AA subjects (mean age, 42 years [SD, 8.9]; 1523 women [40.9%]) were included in the analysis (2596 from Yale-Penn-1 and 1128 from Yale-Penn-2). There were 173 subjects (4.6%) with partially missing ND or MD criteria, which were imputed. Among subjects with imputed phenotype data, the average number of items that needed to be imputed was 1.2 for ND and 2.1 for MD. For ND, the imputation correlation was 0.74 for Yale-Penn-1 and 0.71 for Yale-Penn-2; for MD, the imputation correlation (between imputed phenotypes and their true hidden values) was 0.86 for both datasets. The median comorbid criterion count was 8.1 (interquartile range [IQR], 4.5–12.5) (Table 1). The distributions of comorbid criterion counts are shown in Figure S1.

**Table 1:** Demographic characteristics of the samples

	Yale-Penn-1	Yale-Penn-2	Total
Sample size (female %)	3227 (47.1)	1717 (41.7)	4944 (45.2)
*GWAS*			
Tobacco exposed (female %)	2596 (44.3)	1128 (33.2)	3724 (40.9)
Age, mean (SD), years	41.5 (8.2)	42.0 (10.4)	41.7 (8.9)
Subjects with partial missing (%)	121 (4.7)	52 (4.6)	173 (4.6)
–with partial missing of ND	20	11	31
–with partial missing of MD	101	41	142
Imputation correlation of ND	0.74	0.71	
Imputation correlation of MD	0.86	0.86	
Median (IQR) of ND+MD	7.9 (4.5–12.5)	8.8 (3.8–12.4)	8.1 (4.5–12.5)
Median (IQR) of ND	5 (3–6)	4 (3–6)	5 (3–6)
Median (IQR) of MD	5 (0–8)	6 (0–8)	5 (0–8)
Correlation between ND and MD	0.15	0.20	0.16

*ND* nicotine dependence, *MD* major depression, *IQR* interquartile range

### Genome-wide significant association

**Fig. 1:**
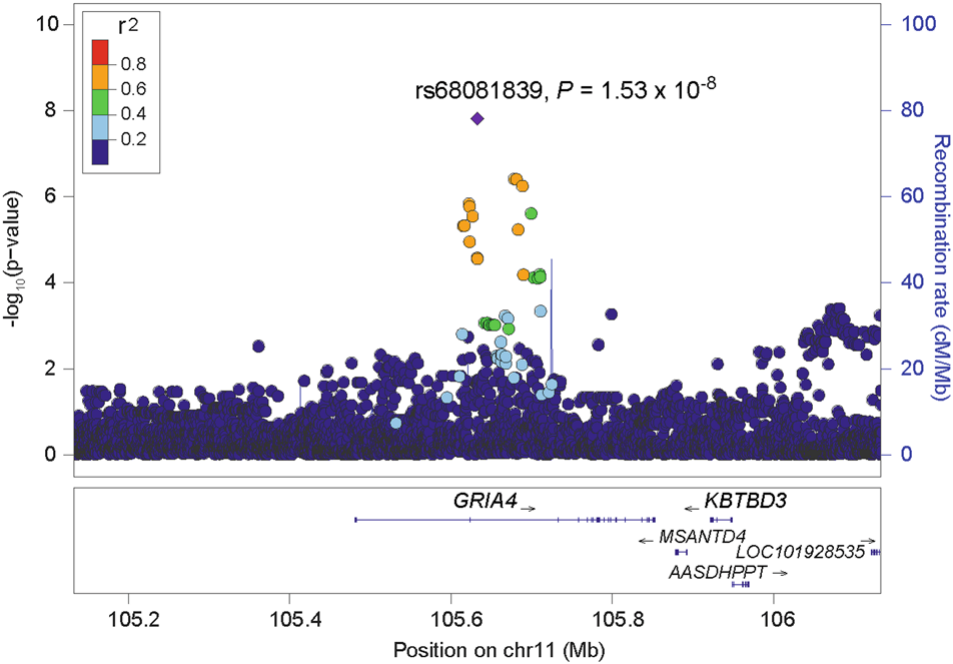
Regional Manhattan plot of rs68081839

GWAS was performed in each dataset, followed by meta-analysis (Figure S2; SNVs with *P*-values < 1 × 10^−4^ (in either individual sample or the meta-analysis) are listed in Table S1). No GWS signals were detected in either sample analyzed individually. In the meta-analysis, a significant association was detected in *GRIA4* (rs68081839, a single nucleotide deletion, −/T; the frequency of the risk allele (−) is 0.68, beta coefficient [*β*] = 0.69 [95% CI, 0.43–0.89], *P* = 1.53 × 10^−8^, Fig. 1). This variant was well imputed in both Yale-Penn-1 (INFO = 0.91) and Yale-Penn-2 (INFO = 0.87) samples. rs68081839 was nominally associated in both the Yale-Penn-1 (*P* = 1.17 × 10^−5^) and Yale-Penn-2 (*P* = 2.95 × 10^−4^) samples.

### Conditional analyses of rs68081839

**Fig. 2:**
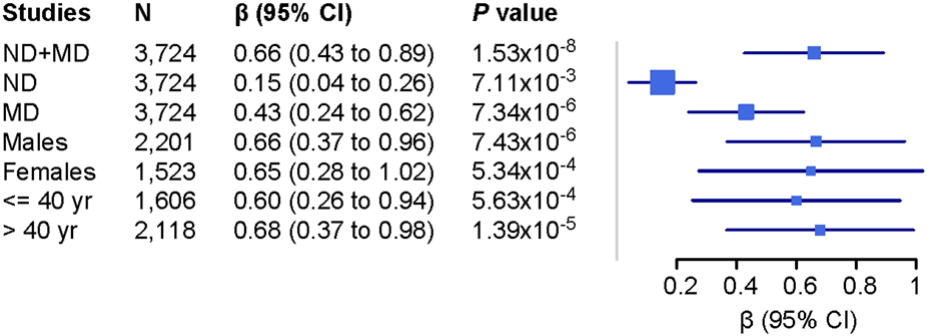
Conditional analysis of rs68081839 and associations in different groups. Association with ND was adjusted for MD; then, association with MD was adjusted for ND

We tested the association of rs68081839 with FTND scores (controlling for MD criterion counts) and MD criterion counts (controlling for FTND scores) to determine whether the association was being driven by a single disorder. Both traits were nominally associated with rs68081839 (*P* = 7.11 × 10^−3^ for ND and *P* = 7.34 × 10^−6^ for MD), indicating an additive or synergistic association for ND–MD comorbidity: i.e., the risk allele contributes to the risk of each trait taken individually. To test whether the association effect was age- or sex-related, we split the sample into older (>40 years old) and younger groups (≤40), adjusting for sex and 10 PCs, and into male and female groups, adjusting for age and 10 PCs. Similar associations between rs68081839 and ND+MD were observed with each of these approaches, indicating a consistent effect in all of the subgroups (Fig. 2). We also tested whether rs68081839 has pleiotropic effects with other substance dependence traits including alcohol, cocaine, marijuana, and opioids, and found no evidence for association (all *P*-values >0.5).

### Functional assessment of GRIA4

*GRIA4* codes for subunit 4 of the AMPA glutamate receptor and it is implicated in glutamate signaling and neuroplasticity^46,47^. It is involved in several KEGG pathways (e.g., amphetamine addiction, nicotine addiction, the cAMP signaling pathway, neuroactive ligand–receptor interaction, glutamatergic synapses, dopaminergic synapses^48^). We explored the gene expression profiles of *GRIA4* in different tissues from GTEx^41^, where it is shown to be widespread and primarily expressed in human brain (Figure S3). We then evaluated the spatio-temporal transcriptome of *GRIA4* in human brain^42^. High expression of *GRIA4* across several brain regions was observed in adulthood, increasing from the early fetal periods (Figure S4). The consistent high level of expression in brain supports the functional relevance of *GRIA4* in psychiatric traits.

To investigate the functional relevance of *GRIA4* further, the top 100 genes co-expressed with *GRIA4* were derived from COXPRESSdb^43^ (Table S2). These include *NLGN1*, *KCND2*, *ELAVL4*, *NXPH1*, *GRM5*, and *GABRB1*. We assessed the disease enrichment of the co-expressed genes using web-based tool WebGestalt^44^ and found that mental disorders, depression, bipolar disorder, and anxiety disorder were significantly enriched (FDR < 0.05, Table S3).

### Gene ontology enrichment analysis

The top SNVs (*P* < 1 × 10^−4^) were mapped to 223 genes (Table S1). GO enrichment analysis using the GOrilla web tool^45^ showed these genes to be enriched for cell adhesion, calcium ion binding, synapse, and plasma membrane (Table 2). We also tested the tissue expression enrichment using DAVID^49,50^; it showed significant enrichment in the brain (*P* = 5.44 × 10^−6^, FDR = 6.29 × 10^−3^). Disease enrichment analysis using WebGestalt showed that the top genes are enriched in various psychiatric disorders including bipolar disorder, anxiety disorder, depression, and substance-related disorders (Table S4). Taken together, the enrichments in signal transduction, synapse, and mental disorders support the interpretation that the polygenic risk of ND+MD is related to neural functions.

**Table 2:** Gene ontology enrichment of the genes mapped to top SNVs (*P* < 1 × 10^−4^)

Category	Term	*P*	FDR
Biological process	GO:0098742—cell–cell adhesion via plasma-membrane adhesion molecules	1.81 × 10^−12^	2.67 × 10^−8^
	GO:0007156—homophilic cell adhesion via plasma membrane adhesion molecules	6.14 × 10^−11^	4.52 × 10^−7^
	GO:0098609—cell–cell adhesion	4.14 × 10^−8^	2.03 × 10^−4^
	GO:0007155—cell adhesion	5.98 × 10^−8^	2.20 × 10^−4^
	GO:0022610—biological adhesion	6.52 × 10^−8^	1.92 × 10^−4^
Molecular function	GO:0005509—calcium ion binding	1.43 × 10^−6^	6.29 × 10^−3^
Cellular component	GO:0045202—synapse	1.60 × 10^−6^	2.89 × 10^−3^
	GO:0044459—plasma membrane part	2.67 × 10^−6^	2.42 × 10^−3^
	GO:0005887—integral component of plasma membrane	2.05 × 10^−5^	1.24 × 10^−2^
	GO:0031226—intrinsic component of plasma membrane	2.23 × 10^−5^	1.01 × 10^−2^
	GO:0005886—plasma membrane	8.50 × 10^−5^	3.08 × 10^−2^
	GO:0031224—intrinsic component of membrane	8.92 × 10^−5^	2.69 × 10^−2^

## Discussion

ND and MD are among the most common psychiatric disorders worldwide and are associated with substantial morbidity and mortality^51^. The association between ND (as well as smoking) and MD has been well established, and both shared and distinct etiologies have been postulated. GWAS have identified risk or protective variants for ND and MD individually. To our knowledge, this is the first study of the shared genetic risks for ND and MD comorbidity. To accomplish this, we employed a dimensional approach using our phenotype data collected using the SSADDA. We found one SNP to be significantly associated with ND+MD comorbidity (*β* = 0.69 [95% CI, 0.43–0.89], *P* = 1.53 × 10^−8^, Fig. 1). rs68081839 is a single nucleotide deletion in the *GRIA4* gene. Conditional analyses showed that the association was not driven by ND or MD alone; instead, there is an additive or synergistic effect of ND and MD. In our dataset, the contribution from MD is greater than that from ND (Fig. 2). There was no evidence of pleiotropy with other substance dependence traits (based on direct testing for association).

*GRIA4* (glutamate ionotropic receptor AMPA type subunit 4)—also referred to as GluR-D or GluR4—is a member of the AMPA-selective glutamate receptor family (AMPARs). AMPARs are expressed ubiquitously in the central nervous system and are the predominant excitatory neurotransmitter receptors in the mammalian brain. They are localized at the postsynaptic membrane and are essential for synaptic plasticity^52^. The most thoroughly characterized examples of synaptic plasticity are long-term potentiation (LTP) and long-term depression (LTD), widely believed to be the cellular basis of learning and memory^53^. Further studies have shown that LTP and LTD participate in pathological processes such as Alzheimer’s disease, schizophrenia, and addiction^52,54^. *GRIA4* functions as a ligand-gated ion channel in the central nervous system and plays an important role in fast excitatory synaptic transmission^46^. Expression of *GRIA4* is sufficient to alter the signaling requirements for LTP during a critical period of synapse development^47^, and the membrane proximal region of *GRIA4*, needed for receptor trafficking and synaptic plasticity, is essential for long-term fear memory formation^55^.

Changes in *GRIA4* expression have been associated with both depression and stress. Postmortem studies showed *GRIA4* upregulation in depressed patients. Expression of *GRIA4* in Brodmann area 10 and amygdala was increased in subjects who died by suicide during an episode of MD compared to subjects who died by suicide without depression, or controls who died suddenly from other causes and had no history of suicidal behavior^56^. Higher expression of *GRIA4* in the dorsolateral prefrontal cortex in female patients with MD than that in female controls has been reported^57^. In relation to stress, *Gria4* was upregulated in the hippocampus in stressed rats, and this could be reversed by the antidepressant drug venlafaxine. *Gria4* expression was also increased by chronic treatment with corticosterone, the major stress hormone^58^. An opposite effect was observed in the ventral (but not dorsal) hippocampus in rats which were treated by neonatal handling^59^.

Synaptic plasticity is known to play a key role in drug addiction. Indeed, addiction has been conceptualized as a pathological form of learning and memory, as they share synaptic plasticity mechanisms. Synaptic plasticity may contribute to different aspects of addiction, including craving, withdrawal, and relapse^60,61^. Altered expression of *GRIA4* and other glutamatergic genes in postmortem hippocampus was observed after chronic exposure to alcohol or cocaine^62^, perhaps contributing to the development of craving^63^. Studies in mouse models showed that AMPARs and N-methyl-D-aspartate receptors (NMDAR) in the ventral tegmental area (VTA) are involved in behavioral sensitization, thus playing key roles in the development of addiction^64,65^. For example, a single exposure to cocaine in vivo can increase the AMPAR/NMDAR ratio in the VTA, which may be involved in an early stage of drug addiction^66^. Along with addictive substances such as cocaine^64^ and morphine^65^, nicotine activates nicotinic acetylcholine receptors (nAChRs) in the VTA to reinforce smoking behavior^67,68^. We therefore speculate that the synaptic plasticity effects of *GRIA4* may explain its contribution to the risk of ND–MD comorbidity.

Besides the significant finding at *GRIA4*, we performed enrichment analyses taking the genes identified by top SNPs (*P* < 1 × 10^−4^) as a whole. The enriched GO terms include cell adhesion in biological processes, calcium ion binding in molecular function, and synapse and plasma membrane in cellular component (Table 2). A significant enrichment of tissue expression was reported in DAVID using the same list of genes. Disease-level enrichment is more informative than GO level enrichment, in this instance: the disease enrichment analyses for the top genes or top coexpressed genes with *GRIA4* shows that the significant enriched disease traits are mainly related to mental disorders (Table S3 and S4). All the reported terms are significant after multiple testing correction (FDR < 0.05). In GWAS with limited sample size, it is very common that no significant enrichment can be detected or the enriched terms cannot be linked to the study trait in an obvious way. Here, despite the sample size limitation, we observed consistent GO or disease enrichments using different web-based tools or different gene lists, indicating that the nominally significant findings (*P* < 1 × 10^−4^) and the coexpressed genes with *GRIA4* are robustly related to the ND+MD trait.

This study has important limitations including modest sample size and the lack of a replication sample. Further studies to understand the biological mechanisms of the genetic risk loci we identified are also warranted.

In conclusion, we identified variation at *GRIA4*, a gene that codes for an AMPA glutamate receptor subunit, as a genetic risk factor for ND and MD comorbidity. This provides initial evidence that variation in the glutamatergic system may underlie the common etiology of these highly comorbid disorders. Thus, the glutamatergic system may thus be a target for treatment of ND^69^, as it is already for MD^70^; especially so in clinical contexts where the two traits are comorbid.

## Electronic supplementary material

Supplemental legends

Supplemental Table S1

Supplemental Table S2

Supplemental Table S3

Supplemental Table S4

Supplemental Figure S1

Supplemental Figure S2

Supplemental Figure S3

Supplemental Figure S4
