# Supplementary material for: Genome-wide association study identifies glutamate ionotropic receptor GRIA4 as a risk gene for comorbid nicotine dependence and major depression
Source: Transl Psychiatry. 2018 Oct 4;8:208. doi: 10.1038/s41398-018-0258-8 (PMC6172277; doi:10.1038/s41398-018-0258-8)
Supplement: Supplementary file 1 — Supplemental legends [file 41398_2018_258_MOESM1_ESM.docx]

**Table S1. Top findings in Yale-Penn-1, Yale-Penn-2 and meta-analysis (*P* < 1 x 10^-4^).** A1 is the effect allele, A2 is non-effect allele, EAF is effect allele frequency.

**Table S2. Top 100 genes co-expressed with *GRIA4*.**

**Table S3. Enriched PharmGKB diseases of the co-expressed genes with *GRIA4*.**

**Table S4. Enriched PharmGKB diseases of the genes mapped to top SNVs (*P* < 1 x 10^-4^).**

**Figure S1. Distribution of comorbid criterion counts.**

**Figure S2. Manhattan and QQ plots.**

**Figure S3. Gene expression of *GRIA4* from GTEx.**

**Figure S4.** **Spatio-temporal transcriptome of *GRIA4* in human brain.** Regions with at least 20 timepoints are presented. RPKM, reads per kilobase per million mapped reads; HIP, hippocampus (hippocampal formation); STR, striatum; MD, mediodorsal nucleus of thalamus; A1C, primary auditory cortex (core); AMY, amygdaloid complex; CBC, cerebellar cortex; DFC, dorsolateral prefrontal cortex; IPC, posteroventral (inferior) parietal cortex; ITC, inferolateral temporal cortex (area TEv, area 20); M1C, MFC, primary motor cortex (area M1, area 4); OFC, orbital frontal cortex; S1C, primary somatosensory cortex (area S1, areas 3,1,2); STC, posterior (caudal) superior temporal cortex (area 22c); V1C, primary visual cortex (striate cortex, area V1/17); VFC, ventrolateral prefrontal cortex.
