# Supplementary figures and images for: Genome-wide association study identifies glutamate ionotropic receptor GRIA4 as a risk gene for comorbid nicotine dependence and major depression
Source: Transl Psychiatry. 2018 Oct 4;8:208. doi: 10.1038/s41398-018-0258-8 (PMC6172277; doi:10.1038/s41398-018-0258-8)

**Yale-Penn-1**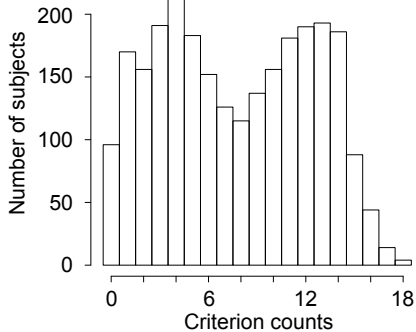**Yale-Penn-2**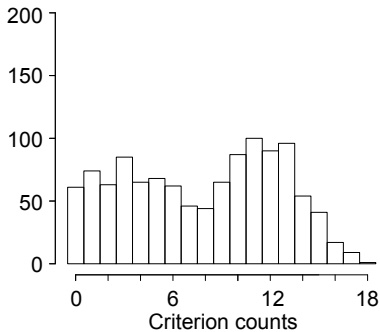

Supplement: Supplementary file 2 — Supplemental Table S1 [file 41398_2018_258_MOESM2_ESM.pdf]

**Yale-Penn-1**

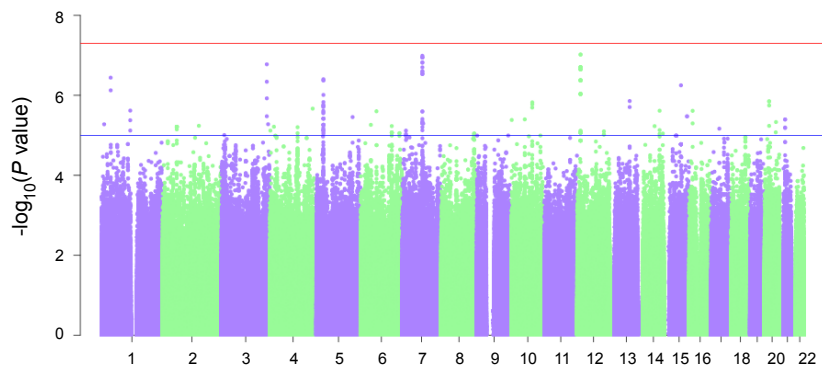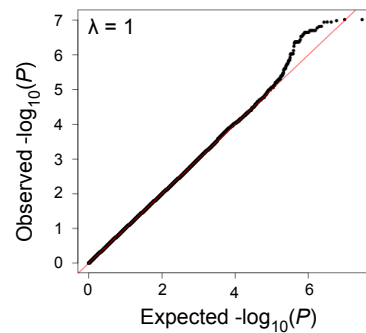

**Yale-Penn-2**

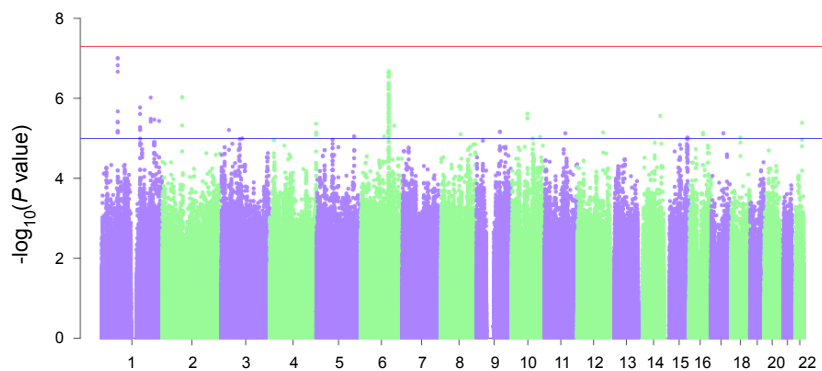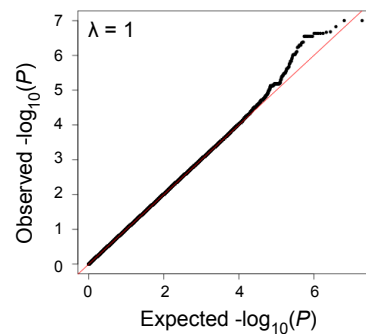

**Meta-analysis**

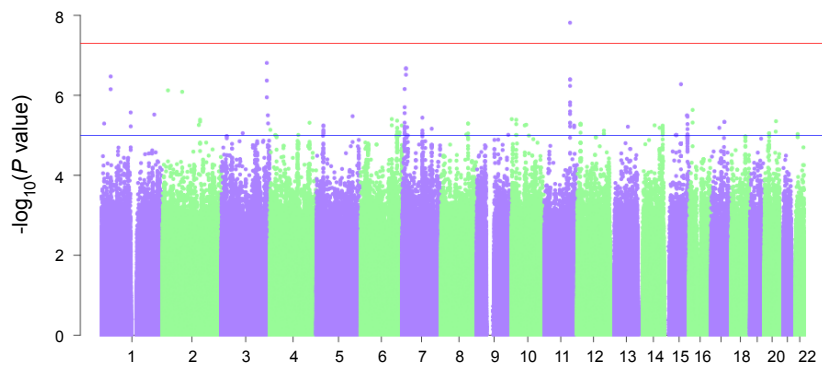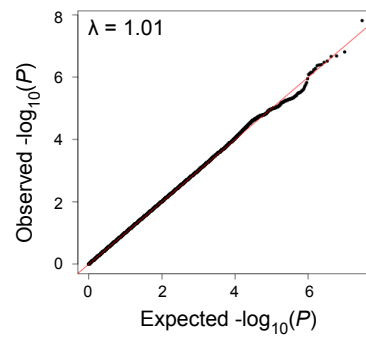

Supplement: Supplementary file 3 — Supplemental Table S2 [file 41398_2018_258_MOESM3_ESM.pdf]

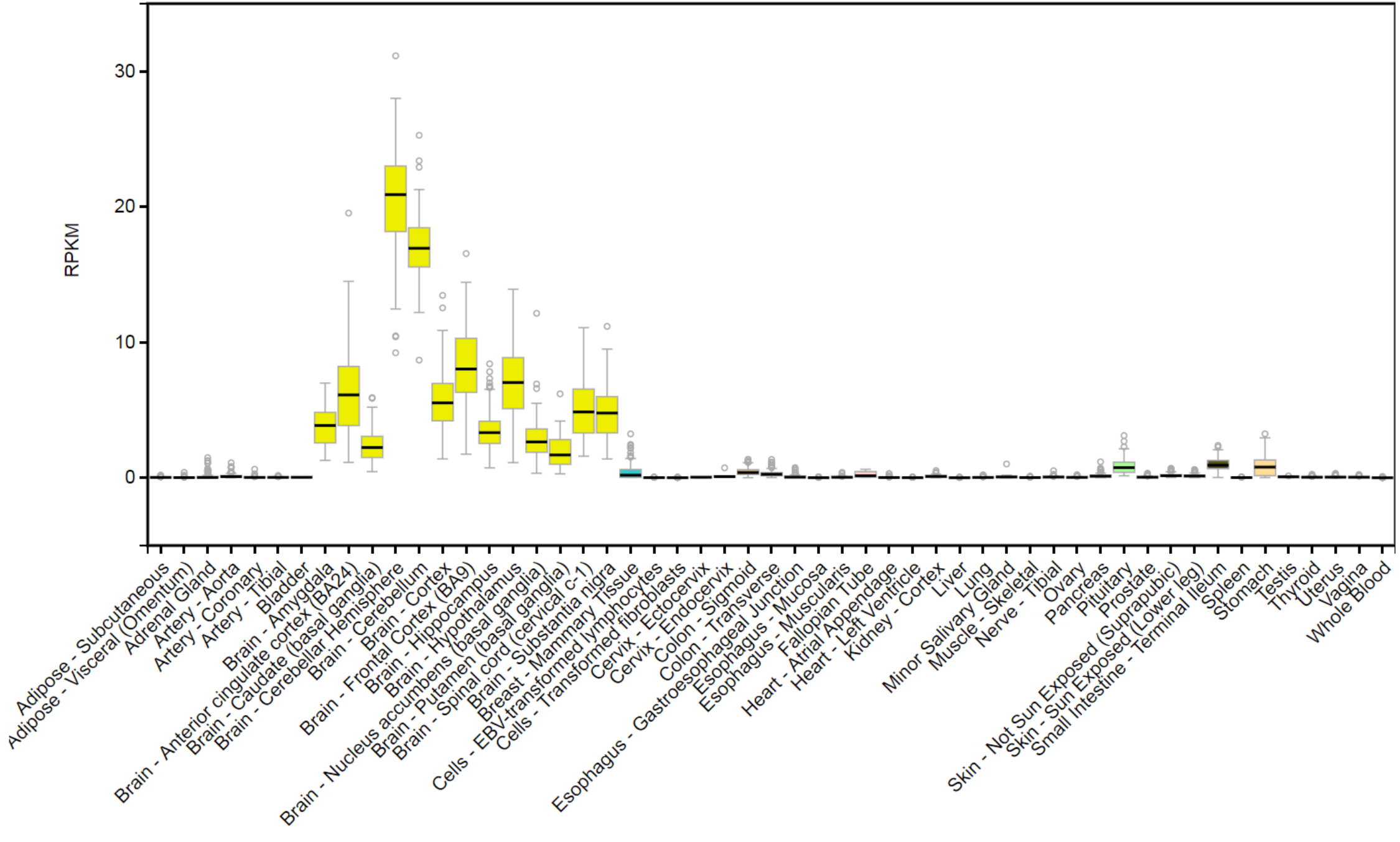

Supplement: Supplementary file 4 — Supplemental Table S3 [file 41398_2018_258_MOESM4_ESM.pdf]

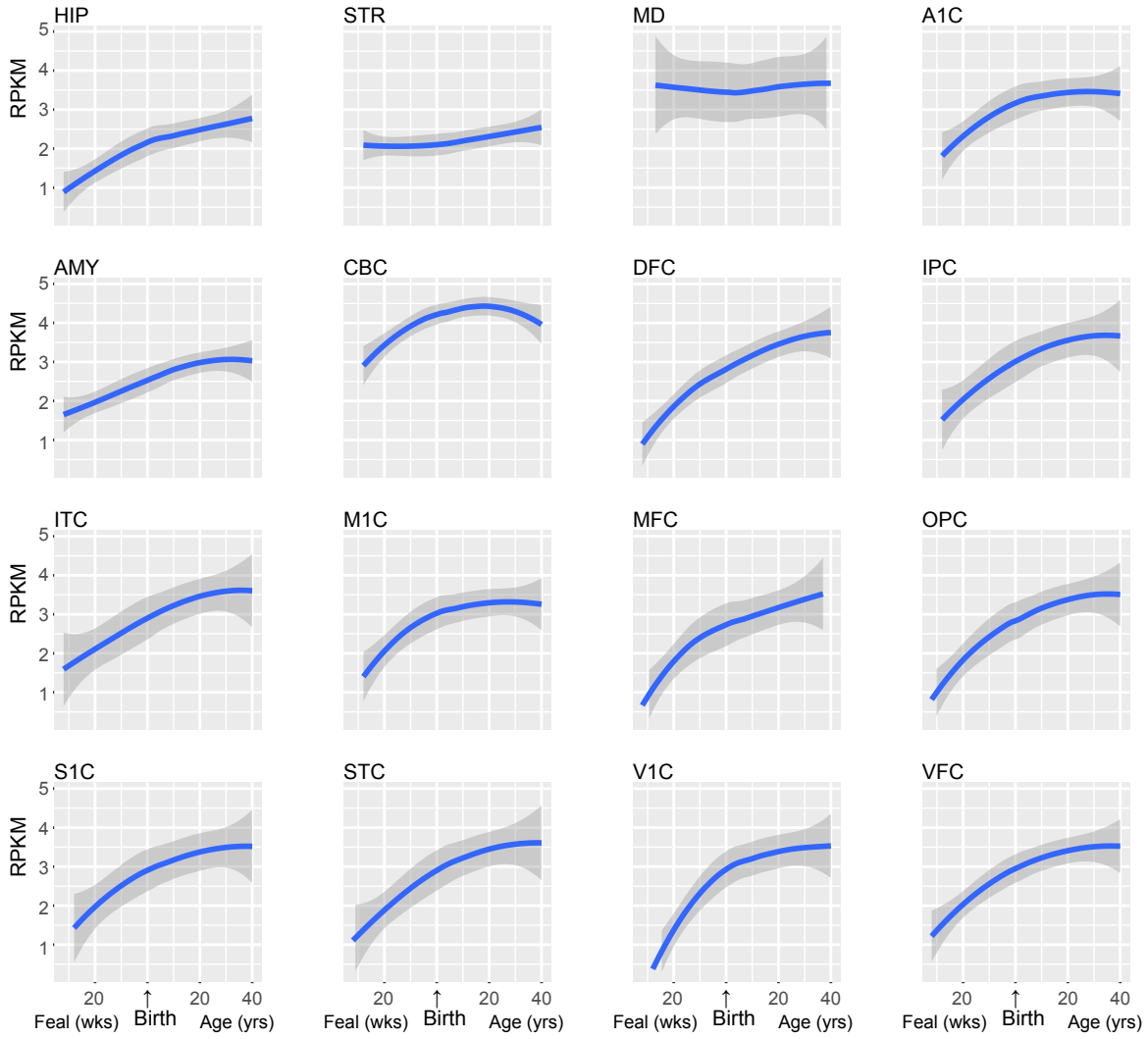

Supplement: Supplementary file 5 — Supplemental Table S4 [file 41398_2018_258_MOESM5_ESM.pdf]
